# INVESTIGATING FORMAL SERVICE UTILIZATION IN CAREGIVERS OF PERSONS LIVING WITH DEMENTIA

**DOI:** 10.1093/geroni/igad104.0465

**Published:** 2023-12-21

**Authors:** Francesca Falzarano, Rachel Bloom, Megan McCarthy, Francesco Osso

**Affiliations:** Weill Cornell Medicine, New York City, New York, United States; Fordham University, New York City, New York, United States; Weill Cornell Medicine, New York City, New York, United States; Weill Cornell Medicine, New York City, New York, United States

## Abstract

In the United States, no acceptable alternatives exist to the heavy reliance on family caregivers in maintaining the long-term services and supports system. Despite their vital role, caregivers of persons living with dementia (PLWD) are often overlooked in healthcare encounters and their needs remain unaddressed, which can lead to deleterious impacts on the caregiver’s health, well-being, and capacity to provide adequate care. Although formal services and resources to assist caregivers of PLWD exist, there is a concerning disconnect between referral and service use uptake – largely due to a siloed, bureaucratic health system in addition to caregivers’ lack of knowledge, awareness, applicability, and access. However, the individual and psychosocial factors that influence attitudes toward and utilization of services remain unclear. Thus, the purpose of this study was to examine correlates of formal service use using data from an NIA-funded mixed-methods study of 46 caregivers of PWLD (Mage=55.9, SD=16.3). Across all domains queried (e.g., home health aides, adult day programs) at least 50% of the sample reported lack of use. Caregivers cited concerns regarding lack of trust, safety of the care-recipient, lack of flexibility, and cost as service barriers. Spousal, compared to adult-child, caregivers had more negative perceptions regarding formal services. Further, greater functional dependency, higher pre-loss grief, and lower levels of self-efficacy and preparedness were associated with more negative attitudes toward service use. These results underscore the importance in ascertaining the holistic needs of caregivers to inform more tailored efforts to connect caregivers’ unique needs to relevant services.

